# Editorialets

**Published:** 1911-01

**Authors:** 


### Editorialets.

Dr. C. F. Taylor, editor of the *Medical World*, Philadelphia, is doing excellent missionary work in his "Monthly Talks to Doctors." They are of practical value, and no doubt save many a doctor from being swindled by the G. R. Q. concerns. In his November issue he had also a powerful editorial on the organized opposition to a Department of Public Health. The *World* is one of my most highly prized exchanges. Every issue is worth a year's subscription. Subscription, $1.00. Add it to your list, Doctor.

---

Dr. Wm. Osborne has removed from Bluffton, Texas, to Greenville, Texas.

---

Lovelady, Texas, December 27, 1910.

*Dear Dr. Daniel:*

For the twenty-sixth time I enclose a piece of money to pay for the "Red Back," which has always proven itself to be a "needed help." Wishing you and yours many returns of this great international anniversary and especially a good year during 1911, I am, Yours truly,

R. W. Skipper.

COLQUITT'S APPOINTMENTS.—*Texas State Board of Medical Examiners.*—Governor Colquitt has appointed the following members of the old Board: Collins and Osborne, "Regular"; Bailey, Osteopath; Daniel, of Honey Grove, Eclectic; Mitchell, Eclectic; Braswell, Physio-Med.; Crowe, Homeopath, and has added Crossthwaite and J. H. Evans, "Regular"; Barber of Wood,—persuasion, unknown to yours truly, and P. M. Peck, Osteopath.

*State Board of Health.*—Dr. Ralph Steiner, of Austin, President; Drs. A. W. Fly, Galveston; B. M. Worsham, El Paso; H. L. McLaurin, Dallas; Beall, of Fort Worth; Herff, of San Antonio, and H. D. Barnes, Tulia, Texas.

The Asylum Superintendents are: Dr. Jno. Preston, Austin, reappointed; Dr. F. S. White, Southwestern Asylum, San Antonio; W. D. Powell, North Texas Asylum, Terrell; T. B. Bass, Epileptic Colony, Abilene. Dr. W. B. McGlisson, Quarantine Officer at Galveston.

Dr. J. S. Abbott has been reappointed Pure Food Commissioner; Dr. Van E. McFarland reappointed Quarantine Officer at Eagle Pass; Dr. A. S. Pollock appointed Quarantine Officer at Sabine Pass; Dr. B. H. Carleton, Quarantine Officer at Velasco.

---

DR. M. M. CARRICK, Assistant Superintendent Epileptic Colony at Abilene, was elected President Taylor County Medical Society on December 6th (ult.).

---

DR. S. E. HUDSON was elected President Travis County Medical Society, and Dr. T. J. Bennett, President Austin District (Seventh) Medical Society.

---

DR. A. L. LINCECUM, of El Campo, was elected President of the Eighth District Medical Society on October 27th.

---

IN GIVING CREDIT, last month, to Drs. Sharp, Carrington, Bogart, Bellfield et al., for pioneer work in efforts to arrest the propagation of defective humanity, I failed to mention Lydston, for which I apologize. As early as 1905, he advocated asexualization, in his powerful work on "Diseases of Society." Ochsner also wrote even prior to Lydston.

